# Do parental heights influence pregnancy length?: a population-based prospective study, HUNT 2

**DOI:** 10.1186/1471-2393-13-33

**Published:** 2013-02-05

**Authors:** Kirsti Myklestad, Lars Johan Vatten, Elisabeth Balstad Magnussen, Kjell Åsmund Salvesen, Pål Richard Romundstad

**Affiliations:** 1 Department of Public Health, Faculty of Medicine, Norwegian University of Science and Technology, Trondheim N-7489, Norway; 2 Department of Obstetrics, Children`s and Women`s Health, Trondheim University Hospital St. Olav, Olav Kyrres gt.11, Trondheim N-7006, Norway; 3 Department of Laboratory Medicine, Children`s and Women`s Health, Norwegian University of Science and Technology, Trondheim N-7491, Norway; 4 Clinical Sciences, Department of Obstetrics and Gynecology, Lund University, Lund, Sweden

**Keywords:** Cardiovascular risk factors, Maternal height, Paternal height, Pregnancy length, Pre-term birth

## Abstract

**Background:**

The objective of this study was to examine the association of maternal and paternal height with pregnancy length, and with the risk of pre- and post-term birth. In addition we aimed to study whether cardiovascular risk factors could explain possible associations.

**Methods:**

Parents who participated in the Nord-Trøndelag Health Study (HUNT 2; 1995–1997) were linked to offspring data from the Medical Birth Registry of Norway (1997–2005).

The main analyses included 3497 women who had delivered 5010 children, and 2005 men who had fathered 2798 pregnancies. All births took place after parental participation in HUNT 2. Linear regression was used to estimate crude and adjusted differences in pregnancy length according to parental heights. Logistic regression was used to estimate crude and adjusted associations of parental heights with the risk of pre- and post-term births.

**Results:**

We found a gradual increase in pregnancy length by increasing maternal height, and the association was essentially unchanged after adjustment for maternal cardiovascular risk factors, parental age, offspring sex, parity, and socioeconomic measures. When estimated date of delivery was based on ultrasound, the difference between mothers in the lower height quintile (<163 cm cm) and mothers in the upper height quintile (≥ 173 cm) was 4.3 days, and when estimated date of delivery was based on last menstrual period (LMP), the difference was 2.8 days. Shorter women (< 163 cm) had lower risk of post-term births, and when estimated date of delivery was based on ultrasound they also had higher risk of pre-term births. Paternal height was not associated with pregnancy length, or with the risks of pre- and post-term births.

**Conclusions:**

Women with shorter stature had shorter pregnancy length and lower risk of post-term births than taller women, and when EDD was based on ultrasound, they also had higher risk of preterm births. The effect of maternal height was generally stronger when pregnancy length was based on second trimester ultrasound compared to last menstrual period. The association of maternal height with pregnancy length could not be explained by cardiovascular risk factors. Paternal height was neither associated with pregnancy length nor with the risk of pre- and post-term birth.

## Background

The mechanisms that trigger onset of delivery and determine pregnancy length are poorly understood. Offspring sex, fetal growth, parity, and genetic factors may influence biological variation of pregnancy length, and various obstetric complications, including maternal and fetal disease may shorten length of pregnancy [1-4]. It is not clear if maternal height is a determinant of the length of pregnancy, and studies of maternal height and pregnancy length have shown conflicting results [5,6].

Nonetheless, it has been suggested that women of short stature are at increased risk of preterm births and other pregnancy complications that may shorten pregnancy length [5,7-13]. Short women are also at increased risk of cardiovascular disease later in life, and an unfavorable cardiovascular risk profile prior to pregnancy (increased blood pressure and body mass index (BMI), and unfavorable serum lipids and glucose) have been associated with increased risk of preterm birth, low offspring birth weight and preeclampsia [14-17]. Thus, factors associated with cardiovascular risk may underlie a possible association between maternal height and pregnancy length.

Among men, short stature is also associated with increased risk of cardiovascular disease [17], but in contrast to the risk among women [14], it is not known whether unfavorable and common paternal cardiovascular risk factors may influence length of the fathered pregnancy. Neither is it known whether paternal height per se is associated with length of pregnancy. Still, results of large intergenerational studies suggest that paternal factors may contribute to the variation in pregnancy length [1,2,18,19].

Therefore, the main aims of this study were to assess maternal and paternal height in relation to pregnancy length, and to examine whether parental height is associated with risk for pre- or post-term birth. We have also assessed whether any association of parental height could be attributed to cardiovascular risk factors, and whether common paternal cardiovascular risk factors, including blood pressure, BMI, serum glucose and lipids, are associated with pregnancy length.

## Methods

Data on parental height and cardiovascular risk factors were retrieved from the second wave of a large population based study in Norway (HUNT 2) that was conducted between 1995 and 1997. By individual linkage to the Medical Birth Registry of Norway, we identified all births that occurred between 1997 and 2005 and had been parented by participants in the HUNT 2 Study.

The primary aim of the HUNT 2 Study was related to major public health issues, such as cardiovascular disease, diabetes, obstructive lung disease, osteoporosis and mental health. The HUNT 2 Study has been described in detail elsewhere [20]. Briefly, all residents of Nord-Trøndelag County 20 years or older were invited, and 66 140 of the 94 194 eligible adults (71%) attended the study, that included a clinical examination carried out by specially trained nurses and technicians. Standardized measurements of height, weight, waist circumference, blood pressure, and non-fasting serum lipids and glucose were performed. Additionally, the participants responded to a comprehensive medical and life style-related questionnaire.

Body height was measured to the nearest 1.0 cm and weight to the nearest 0.5 kg. Body-mass index (BMI) was calculated as weight in kilograms (kg) divided by the squared value of height in meters (m2). Blood pressure measurements were repeated three times with one minute intervals and measured by an automatic oscillometric method (Dinamap 845 XT; (Critikon, Tampa, Florida). The mean of the second and third measurements was recorded and cuff size was adjusted to arm circumference. Venous blood sampling was done non-fasting at attendance, but with the registration of time since last meal. Serum lipids were analyzed on a Hitachi 911 Auto analyzer (Hitachi,Mito,Japan) with reagents from Boehringer Mannheim (Mannheim,Germany). Total cholesterol and HDL cholesterol were measured by an enzymatic colorimetric cholesterolerase method, and HDL cholesterol was measured after precipitation with phosphortungsten and magnesium ions. Triglycerides were also measured with an enzymatic colorimetric method, and glucose was measured by an enzymatic hexokinase method.

All live- and stillbirth after 16 weeks of gestation are compulsory notified to the Medical Birth Registry (MBR) of Norway. The registration is based on a standardized form completed by midwives or doctors at the delivery units, and contains information related to maternal health before and during pregnancy, complications of pregnancy and delivery and perinatal data of the newborn.

Last menstrual period (LMP) was used to estimate expected date of delivery (EDD) from 1967 until ultrasound (US) became the national standard dating method in 1999. LMP has still been recorded for all women after 1999 and is used when ultrasound data is missing. In the current study, LMP was available for nearly all births and ultrasound data was available for more than 80% of the births.

In Norway, one routine ultrasound examination is offered to all pregnant women between 17–19 weeks of gestation, and the offer accepted by more than 98% of the women [21]. In 1997–2005 EDD was based on measurement of the outer-outer border of fetal biparietal diameter (BPD). Preterm deliveries were defined as occurring before 259 days (< 37 +0 weeks) of gestation, and post-term pregnancies as lasting 294 days or more (≥ 42 +0 weeks). The proportion of post-term pregnancies will be influenced by hospital guidelines for induction of labor. During the study period, the Norwegian official guidelines recommended assessment of all pregnancies at 296 days (42+2 weeks). Women at high risk of complications were offered induction of labor, whereas low-risk women were subject to expectant management until spontaneous delivery or induction at 43 +0 weeks.

We calculated z-scores of birth weight according to sex and gestational age, using estimated standards from births registered in MBR [22]. Estimates of gestational age were based on LMP [22]. Z-scores indicate the standard deviations of the offspring’s birth weight above or below the expected mean for gestational age and sex. In this study, small for gestational age offspring (SGA) was defined as z-score of birth weight adjusted for gestational age and sex below the 2.5 percentile. Diagnoses of preeclampsia were based upon hospital medical records noted in the MBR, using the following diagnostic criteria: The onset of blood pressure increase to 140/90 mm Hg in combination with proteinuria (protein excretion of ≥ 0.3 grams per 24 hours or ≥ + on dip stick) after 20 weeks of gestational age.

Onset of labour was categorized as either spontaneous or induced. Induction of labour was either by medication or by caesarian section. Induced caesarian sections included all elective sections (planned and performed more than 8 hours after the decision was made), and acute sections (planned and performed less than 8 hours after the decision was made) performed before labour. In 1997–2005 elective caesarian sections in Norway were recommended to be performed around 38 gestational weeks Data on indications for caesarian sections are insufficient in the MBR of Norway.

Among women who participated in HUNT 2, a total of 6 122 births were recorded in MBR (gestational age > 22.0 weeks and birth weight > 425 grams) from 1995 to 2005. Among these births, 1 022 were excluded from the analysis because of either multiple pregnancies (n=252) or possible ongoing pregnancies at the time of data collection (n=770). In addition, 90 were excluded due to missing data on the following variables: maternal height (n=5), information on social benefits (n=49), information on diabetes or chronic hypertension prior to pregnancy (n=7), and information on cardiovascular risk factors prior to pregnancy (n=29). Thus, the main analyses included 5 010 births among 3 497 mothers.

For the paternal analyses, 2 210 of 5 010 pregnancies were excluded because the father did not participate in HUNT 2, and 2 births had missing data on paternal height, thus leaving a total of 2 798 fathered pregnancies among 2 005 men to be analyzed.

### Statistical methods

We used linear regression analyses to estimate crude and adjusted differences in pregnancy length according to parental heights, and logistic regression to estimate crude and adjusted associations of parental heights with the risk of pre- and post-term deliveries. Separate analyses were done for mothers and fathers and for ultrasound and LMP dating. To account for more than one delivery by the same woman, we used the cluster option with robust standard errors in STATA for Windows which is recommended to obtain variance estimates with adjustments for within-cluster correlation [23]. Parental heights were categorized into quintiles in order to evaluate trends across quintiles. Tests for trend across quintiles were performed by scoring categories from 1 to 5, and by including the scores as a continuous variable in the models.

In multivariable analyses we adjusted for potentially confounding factors in three steps. In the first model, we adjusted for the parents’ age at enrollment in the HUNT 2 Study. In the second model, we additionally adjusted for offspring sex, time interval between parental participation in HUNT 2 until birth (continuous), maternal age at delivery (continuous), parity (0, 1, and 2 or more previous births), maternal smoking status in HUNT 2 (no / yes), educational level (< 10 years/ 10–12 years/ > 12 years), and socioeconomic position (employed, student or housewife / unemployed or receiving social security benefits), and partner’s height. In the third model, we additionally adjusted for cardiovascular risk factors of the mother prior to pregnancy; i.e. body mass index (continuous), systolic and diastolic blood pressure (continuous), serum glucose, total serum cholesterol, HDL cholesterol and triglycerides (continuous), prevalent diabetes mellitus, chronic hypertension, and kidney disease.

For fathers, we additionally conducted multivariable analyses of blood pressure, BMI, serum lipids and glucose with pregnancy length.

We evaluated the results of adjusting for intermediate factors that could lie on the causal pathway of the association between maternal height and pregnancy length or, alternatively; could be confounders. Induction of labour (by medication or by caesarian section), and pregnancy complications were regarded as possible intermediate factors. To assess the role of induction, we restricted the main analyses to spontaneous onset of delivery (excluding medically induced deliveries plus all elective caesarian sections plus acute caesarian sections before onset of labour). Separately, we excluded pregnancy complications such as SGA (offspring below the 2.5 percentile), preeclampsia, and stillbirth) in addition to the induced deliveries.

We did linear regression analyses to assess associations between parental height and offspring birth weight in a similar way as was done for pregnancy length. This was done with the purpose of comparing associations between parental height and birth weight, with that of parental height and pregnancy length.

Possible effect modification by cardiovascular risk factors was also assessed in separate analyses. We included an interaction term between maternal height and the cardiovascular risk factor (systolic blood pressure, BMI, glucose, lipids, pregestational metabolic and cardiovascular disease) and a likelihood ratio test was used to compare the fit of models with and without the interaction term.

Stata for Windows (version 12.1, Stata Corporation, College Station, Texas) was used for all statistical analyses. The study was approved by the Norwegian Data Inspectorate, the Norwegian Board of Health, and by the Regional committee for medical research ethics. All the participants of the study assigned an informed consent to participate in the HUNT 2 Study and also approved that data could be linked to the Medical Birth Registry of Norway.

## Results

Descriptive characteristics of the parents participating in HUNT 2 are presented in Table 1. Mean maternal age at participation was 25.9 years. Pregnancy length was associated with maternal age at delivery, maternal smoking and maternal systolic blood pressure, whereas no association with pregnancy length was found for parity, maternal educational status, maternal BMI, paternal BMI and paternal systolic blood pressure (Table 1).

**Table 1:** **Associations of maternal and paternal covariates**^**1 **^**with pregnancy length**^**2 **^**assessed as differences in days**

**Prepregnancy parental covariate**	**Number of births**	**Difference in gestational length(days)**	**95% CI**	**P for trend**
Maternal age at delivery (per 5-years)	4038	−0.5	−1.1, 0.0	0.04
Parity^2^				0.46
0	1107	0	Ref	
1	1502	0.5	−0.5,1.5	
2 or more	1429	−0.4	−1.4,0.7	
Maternal education^3^				0.19
< 10 years	190	0	ref	
10-12 years	2214	3.6	0.7,6.6	
>12 years	1587	3.9	0.9,6.9	
Maternal smoking^4^				0.01
no	2911	0	Ref	
yes	1036	−0.6	−1.6,0.5	
Maternal unemployment and /or receiving social benefits				0.28
No	3305	0	Ref	
Yes	733	−1.1	−1.8,0.5	
Maternal BMI(quintiles)				0.82
≤ 21.3	878	0	Ref	
21.4-22.9	859	0.8		
23.0-24.7	817	0.8		
24.8-27.1	810	1.1		
≥ 27.2	674	−0.1		
Maternal systolic blood pressure, in mm Hg(quintiles)				0.002
< 111	863	0	Ref	
112-117	838	−0.1	−1.4,1.2	
118-122	712	−1.0	−2.4,0.3	
123-130	862	−1.1	−2.5,0.2	
>131	763	−2.0	−3.4,-0.6	
Paternal age at delivery (per 5 years)	2153	−0.1	−0.7,0.6	0.85
Paternal BMI (quintiles)				0.55
≤ 22.9	388	0	Ref	
23.0-24.3	436	2.2	0.3,4.1	
24.4-25.8	445	1.2	−0.8,3.3	
25.9-27.8	463	0.9	−1.2,2.9	
≥ 27.9	421	0.1	−2.1,2.2	
Paternal systolic blood pressure, in mm Hg (quintiles)				0.63
≤122	437	0	Ref	
123-129	399	−1.3	−3.2,0.7	
130-135	440	−0.3	−2.2,1.6	
136-142	428	0.4	−1.5,2.3	
≥143	449	−0.3	−2.1,1.5	

^1^Parental covariates were measured prior to pregnancy, at the HUNT 2 Study, Norway, 1995–1997. ^2^EDD (Expected Date of Delivery) estimated by ultrasound measurement in 17–19 weeks of gestation.^3^Parity = previous births. ^4^Missing data on educational status for 47 women. ^5^Missing data on smoking status for 91 women.

Table 2 describes pregnancy and offspring characteristics, stratified by quintiles of maternal height. Information on LMP was available for nearly 97% of the pregnant women, and 81% had EDD based on ultrasound. Shorter maternal height was associated with lower mean birth weight, lower frequency of spontaneous onset of labour, and higher frequency of onset of labour by caesarian section (elective and acute caesarian sections *before* onset of labour). Shorter women also had higher frequency of total number of caesarian sections, including acute caesarian sections *after* onset of labour. Shorter women had higher rates of SGA offspring below the 2.5 percentile than taller women.

**Table 2:** **Pregnancy and offspring characteristics stratified by maternal height in quintiles**^**1**^

**Pregnancy characteristics**	**Maternal height by quintiles(cm)**					
	**<163**	**163-165**	**166-168**	**169-172**	**>173**	
	**n=1192**	**n=930**	**n=1032**	**n=1053**	**n=803**	**P for trend**
Mean age at delivery, years (SE)	30.3(0.2)	30.5(0.2)	30.3(0.2)	30.2(0.2)	30.6(0.17)	0.83
Mean time from baseline to delivery, years (SE)	4.2(0,1)	4.3(0.1)	4.3(0.1)	4.4(0.1)	4.4(0.1)	0.01
Mean pregnancy length, days, LMP-based (SE)^2^	279.3(0.5)	280.2(0.5)	281.5(0.6)	281.5(0.5)	282.0(0.6)	<0.0001
Mean pregnancy length, days, US-based (SE)^3^	276.7(0.5)	277.9(0.5)	279.2(0.6)	280.1(0.5)	280.9(0.5)	<0.0001
Primiparous mother (%)^4^	327(27)	241(26)	300(29)	309(29)	236(29)	0.06
Mean birth weight, grams (SE)^5^	3490(20)	3618(21)	3655(22)	3692(20)	3803(25)	<0.0001
Male fetal sex (%)	626(52.5)	495(53.2)	531(51.5)	574(54.5)	402(50.1)	0.61
Onset of labour (%)						
Spontaneous	873(73.2)	740(79.6)	835(80.9)	868(82.4)	624(77.7)	0.002
Medically induced	144(12.1)	111(11.9)	137(13.3)	127(12.1)	114(14.2)	0.26
CS before labour^6^	175(14.7)	79(8,5)	60(5.8)	58(5.5)	65(8.1)	<0.0001
CS, total number (%)^7^	302(25)	162(17)	133(13)	100(9.5)	100(12.5)	<0.0001
Acute CS^8^	154(17.9)	64(6.9)	52(5.0)	48(4.6)	49(6.1)	<0.0001
Elective CS^9^	148(12.4)	98(10.5)	81(7.9)	52(4.9)	51(6.4)	<0.0001
SGA < 2.5 percentile (%)^10^	41(3.4)	12(1.3)	18(1.8)	13(1.2)	9(1.1)	0.001
Preeclampsia (%)	50(4.2)	27(2.9)	40(3.9)	31(2.9)	30(3.7)	0.52
Stillbirth (%)^11^	4(0.3)	3(0.3)	4(0.4)	4(0.4)	2(0.3)	0.89

CS = Caesarian section, SE= Standard Error, LMP= last menstrual period, US= ultrasound early second trimester, SGA= small for gestational age.

^1^Data from 5010 singleton pregnancies of 3497 mothers, registered in the Medical Birth Registry of Norway 1995–2005. ^2^Missing data on LMP for 164 pregnancies. ^3^Missing data on US for 972 pregnancies. ^4^Primiparous=woman with no previous birth. ^5^Missing data on birth weight for 1 woman. ^6^Elective and acute caesarian section before labour. ^7^Elective and acute caesarian sections before and after onset of labour. ^8^Planned and performed less than 8 hours after decision was made. ^9^Planned and performed more than 8 hours after decision was made. ^10^Missing data on SGA for 5 pregnancies. ^11^Missing data on stillbirth for 1 woman.

Table 3 shows age-adjusted and multivariable adjusted differences in mean pregnancy length according to quintiles of maternal height. In the age-adjusted analyses gestational age increased by increasing maternal height, and women in the upper quintile (≥ 173 cm) had on average 4.3 days longer pregnancies than women in the reference group (< 163 cm) when EDD was based on ultrasound, and 2.7 days longer when EDD was LMP based. Additional adjustments for obstetric and socioeconomic measures (maternal age at delivery, time between baseline at HUNT 2 and delivery, parity, offspring sex, maternal smoking, educational status, and receiving social benefits or not) did not influence the effect estimates. Neither did additional adjustment for levels of maternal cardiovascular risk factors prior to pregnancy (systolic and diastolic blood pressure, BMI, concentration of glucose and lipids, hypertension, diabetes mellitus, kidney disease and coronary artery disease (Table 3). Additional adjustment for paternal height did not change the results (results not shown).

**Table 3:** **Mean differences in length of pregnancy by maternal height**^**1**^

**Difference in pregnancy length (days)**								
		**Crude estimate**^**2**^	**Adjusted for obstetric and socioeconomic factors**^**3**^			**Additional adjustment for maternal cardiovascular risk factors**^**4**^		
Maternal height (in quintiles)	N	Mean difference	Mean difference	95% CI	p for trend	Mean difference	95% CI	p for trend
Estimated date of delivery based on US								
<163	957	0	0	Ref		0	Ref	
163-165	751	1.3	1.3	−0.1,2.6		1.3	0.0,2.7	
166-168	811	2.6	2.6	1.2,4.0		2.6	1.2,4.0	
169-172	856	3.4	3.4	2.1,4.8		3.4	2.0,4.7	
173+	663	4.3	4.3	2.9,5.6	<0.0001	4.3	3.0,5.7	<0.0001
Estimated date of delivery based on LMP								
<163	1148	0	0	Ref		0	Ref	
163-165	903	1.0	1.0	−0.3,2.4		1.1	−0.2,2.4	
166-168	997	2.3	2.3	0.9,3.7		2.3	0.9,3.7	
169-172	1021	2.2	2.3	1.0,3.6		2.3	1.0,3.6	
173+	777	2.7	2.7	1.3,4.2	<0.0001	2.8	1.3,4.2	<0.0001

^1^Pregnancy length according to estimated date of delivery (EDD) by ultrasound (US) (4038 pregnancies) and by first day of last menstrual period (LMP) (4846pregnancies). ^2^Adjusted for maternal age at baseline (HUNT 2 participation). ^3^Additionally adjusted for maternal age at birth, duration between Hunt 2 participation and delivery, maternal education, parity, maternal smoking, receiving social security benefits or not, and fetal sex. ^4^Additionally adjusted for pre-pregnancy, hypertension, diabetes mellitus, kidney and heart disease, BMI; systolic and diastolic blood pressure, concentration of glucose and lipids.

Table 4 shows age-adjusted and multivariable adjusted differences in pregnancy length according to quintiles of maternal height after restriction of the analysis to births with spontaneous onset of delivery. For deliveries with spontaneous onset, the difference in pregnancy length between the upper and lower maternal height group was reduced from 4.3 days (95% CI 3.0, 5.7) to 3.5 days (95% CI 2.2, 4.8), according to ultrasound dating, and from 2.8 days (95% CI 1.4, 4.2) to 1.9 days (95% CI 0.4, 3.4) according to LMP dating (Table 4). Additional exclusion of pregnancy complications (SGA, preeclampsia, and stillbirth) did not substantially change these results (results not shown).

**Table 4:** **Mean differences in length of pregnancy by maternal height**^**1**^ restricted to pregnancies with spontaneous onset of delivery^**2**^

		**Difference in pregnancy length (days)**						
		**Crude estimate**^**3**^	**Adjusted for obstetric and socioeconomic factors**^**4**^			**Additional adjustment for maternal cardiovascular risk factors**^**5**^		
**Maternal height (in quintiles)**	**N**	**Mean difference**	**Mean difference**	**95% CI**	**p for trend**	**Mean difference**	**95% CI**	**p for trend**
Estimated date of delivery based on US								
<163	687	0	0	Ref		0	Ref	
163-165	593	0.5	0.5	−0.8,1.8		0.5	−0.8,1.8	
166-168	654	1.8	1.9	0.5,3.3		1.9	0.5,3.3	
169-172	703	1.8	1.9	0.5,3.2		1.8	0.4,3.1	
173+	509	3.5	3.6	2.3,4.8	<0.001	3.5	2.2,4.8	<0.001
Estimated date of delivery based on LMP								
<163	850	0	0	Ref		0	Ref	
163-165	723	0.3	0.4	−1.0,1.7		0.4	−1.0,1.8	
166-168	808	1.3	1.4	0.0,2.9		1.4	0.0,2.9	
169-172	839	0.6	0.6	−0.7,2.0		0.6	−0.7,2.0	
173+	611	1.8	1.8	0.4,3.3	0.02	1.9	0.4,3.4	0.02

^1^Pregnancy length according to date of delivery estimated by ultrasound (US) (3146 pregnancies) and by first day of last menstrual period (LMP) (3831 pregnancies). ^2^Exclusion of pregnancies with induced onset of delivery (medically or by caesarean section). ^3^Adjusted for maternal age at baseline (HUNT 2 participation). ^4^Additionally adjusted for maternal age at birth, duration between Hunt 2 participation and delivery, maternal education, parity, maternal smoking, receiving social security benefits or not, and fetal sex. ^5^Additionally adjusted for pre-pregnancy maternal hypertension, diabetes mellitus, kidney and heart disease, BMI; systolic and diastolic blood pressure, concentration of glucose and lipids.

Pregnancies restricted to spontaneous onset of delivery^2^.

Paternal height showed no association with pregnancy length of the partner in age-adjusted and fully adjusted models (Table 5). Neither did we find any association with pregnancy length for common paternal cardiovascular risk factors, including levels of blood pressure, BMI, serum lipids and glucose (results not shown).

**Table 5:** **Mean differences in length of pregnancy by paternal height**^**1**^

		**Difference in pregnancy length (days)**						
		**Crude estimate**^**2**^	**Adjusted for obstetric and socioeconomic factors**^**3**^			**Additional adjustment for maternal cardiovascular risk factors**^**4**^		
**Paternal height (in quintiles)**	**N**	**Mean difference**	**Mean difference**	**95% CI**	**p for trend**	**Mean difference**	**95% CI**	**p for trend**
Estimated date of delivery based on US								
≤ 175	503	0	0	Ref		0	Ref	
176-178	397	0.2	−0.2	−2.0,1.7		−0.5	−2.4,1.4	
179-181	448	−0.1	−0.6	−2.4,1.3		−0.8	−2.6,1.1	
182-185	412	0.6	−0.5	−2.2,1.6		−0.8	−2.7,1.1	
≥186	399	0.5	−0.7	−2.5,1.0	0.43	−0.7	−2.5,1.0	0.39
Estimated date of delivery based on LMP								
≤ 175	642	0	0	Ref		0	Ref	
176-178	496	0.2	0.0	−1.9,1.9		−0.2	−2.0,1.7	
179-181	514	0.2	−0.1	−1.9,1.6		−0.3	−2.0,1.5	
182-185	552	−0.6	−1.2	−3.0,0.7		−1.4	−3.2,0.5	
≥186	512	−0.3	−0.9	−2.7,0.9	0.17	−0.9	−2.7,0.9	0.16

^1^Pregnancy length according to date of delivery estimated by ultrasound (US) (2159 pregnancies) and by first day of last menstrual period (LMP) (2716 pregnancies). ^2^Adjusted for paternal age at baseline (HUNT 2 participation). ^3^Additionally adjusted for maternal height, maternal age at birth, duration between Hunt 2 participation and delivery, maternal education, parity, maternal smoking, receiving social security benefits or not, and fetal sex. ^4^Additionally adjusted for pre-pregnancy hypertension, diabetes mellitus, kidney and heart disease, BMI; systolic and diastolic blood pressure, concentration of glucose and lipids and maternal height.

The risk of post-term deliveries (≥ 42.0 weeks) increased with maternal height (Table 6). In the crude analysis, taller women (≥ 173 cm) had 90% higher odds of post-term pregnancy compared to the reference group when gestational age was estimated by ultrasound (OR 1.9, 95% CI 1.3, 2.9), and 50% higher when gestational age was based on LMP (OR 1.5, 95% CI 1.0,1.9). The risk was slightly increased after adjustment for obstetric factors, socioeconomic measures and cardiovascular risk factors prior to pregnancy. The risk of preterm delivery was lower in taller women when gestational length had been estimated by ultrasound, whereas only weak associations were observed when EDD was determined by LMP.

**Table 6:** **Odds ratio (OR) for preterm delivery and post-term delivery by maternal height**^**1**^

	**Preterm delivery(< 37.0) weeks**					**Post term delivery(>42.0 weeks)**				
**Maternal height (in quintiles)**	**Cases/non-cases**	**Crude estimate**^**2**^	**Adjusted estimate**^**3**^	**95% CI**	**p for trend**	**Cases/non-cases**	**Crude estimate**^**2**^	**Adjusted estimate**^**3**^	**95% CI**	**p for trend**
Estimated date of delivery based on US										
<163	62/895	1.0	1.0	Ref		43/914	1.0	1.0	Ref	
163-165	39/712	0.8	0.8	0.5,1.2		32/719	0.9	1.0	0.6,1.6	
166-168	43/768	0.8	0.8	0.5,1.2		60/751	1.7	1.7	1.1,2.6	
169-172	30/826	0.5	0.5	0.3,0.9		58/798	1.5	1.6	1.1,2.5	
173+	28/635	0.6	0.6	0.4,1.0	0.015	55/608	1.9	2.1	1.4,3.2	<0.0001
Estimated date of delivery based on LMP										
<163	76/1072	1.0	1.0	Ref		112/1036	1.0	1.0	Ref	
163-165	41/862	0.7	0.6	0.4,1.0		91/812	1.0	1.0	0.8,1.4	
166-168	52/945	0.8	0.8	0.5,1.1		143/854	1.5	1.5	1.2,2.0	
169-172	42/979	0.6	0.6	0.4,0.9		129/892	1.3	1.4	1.0,1.8	
173+	45/732	0.9	0.9	0.6,1.3	0.32	105/672	1.5	1.5	1.1,2.0	0.001

^1^Pregnancy length according to date of delivery estimated by ultrasound (US) (4038 pregnancies) and by first day of last menstrual period (LMP) (4846 pregnancies). ^2^Adjusted for maternal age at HUNT participation. ^3^Additionally adjusted for maternal age at birth, duration between baseline (maternal participation in HUNT 2) and delivery, maternal education, parity, smoking, receiving social security benefits, pre-pregnancy maternal hypertension, diabetes mellitus, kidney and heart disease, BMI, systolic and diastolic blood pressure, blood concentration of glucose and lipids.

After restricting the analysis of maternal height with risks of pre- and post-term birth to deliveries with spontaneous onset (Table 7), the effect estimates were not substantially changed in relation to preterm births, but precision was lower due to the fewer numbers of pregnancies. The associations with post-term delivery were slightly weaker for deliveries with spontaneous onset than for unselected deliveries.

**Table 7:** **Odds ratio (OR) for preterm delivery and post-term delivery by maternal height**^**1**^ revertrestricted to pregnancies with spontaneous onset of delivery^**2**^

	**Preterm delivery (< 37.0) weeks**					**Post term delivery (>42.0 weeks)**				
**Maternal height (in quintiles)**	**Cases/non-cases**	**Crude estimate**^**3**^	**Adjusted estimate**^**4**^	**95% CI**	**p for trend**	**Cases/non-cases**	**Crude estimate**^**3**^	**Adjusted estimate**^**4**^	**95% CI**	**p for trend**
Estimated date of delivery based on US										
<163	25/662	1.0	1.0	Ref		22/665	1.0	1.0	Ref	
163-165	18/575	0.8	0.8	0.4,1.5		15/578	0.8	0.8	0.4,1.6	
166-168	17/637	0.7	0.7	0.3,1.3		36/618	1.8	1.8	1.0,3.1	
169-172	18/685	0.7	0.7	0.4,1.3		29/674	1.3	1.4	0.8,2.5	
173+	12/497	0.6	0.6	0.3,1.4	0.18	27/482	1.7	1.9	1.0,3.4	0.01
Estimated date of delivery based on LMP										
<163	42/808	1.0	1.0	Ref		87/763	1.0	1.0	Ref	
163-165	19/704	0.5	0.5	0.3,0.9		71/652	1.0	1.0	0.7,1.4	
166-168	25/783	0.6	0.6	0.3,1.0		109/699	1.4	1.4	1.0,1.9	
169-172	27/812	0.6	0.6	0.4,1.1		94/745	1.1	1.2	0.8,1.6	
173+	25/586	0.8	0.8	0.5,1.4	0.52	76/535	1.3	1.3	0.9,1.9	0.06

^1^Pregnancy length according to date of delivery estimated by ultrasound (3146 pregnancies) and by last menstrual period (3831 pregnancies). ^2^After exclusion of pregnancies with induced onset of delivery (medically or by Caesarean section). ^3^Adjusted for maternal age at HUNT participation. ^4^Additionally adjusted for maternal age at birth,duration between baseline (maternal participation in HUNT 2) and delivery, maternal education, parity, smoking, receiving social security benefits, pre-pregnancy maternal hypertension, diabetes mellitus, kidney and heart disease, BMI, systolic and diastolic blood pressure, blood concentration of glucose and lipids.

Pregnancies restricted to spontaneous onset of delivery^2^.

We found no association between paternal height and risks of pre- or post-term births (Table 8). Paternal height was, however, positively associated with z-score of fetal birth weight (Table 9), and the strength of the association among fathers was similar to the corresponding association for the mothers.

**Table 8:** **Odds ratio (OR) for preterm delivery and post- term delivery by paternal height**^**1**^

	**Preterm delivery (< 37.0) weeks**					**Post term delivery (>42.0 weeks)**				
**Paternal height (in quintiles)**	**Cases/non-cases**	**Crude estimate**^**2**^	**Adjusted estimate**^**3**^	**95% CI**	**p for trend**	**Cases/non- cases**	**Crude estimate**^**2**^	**Adjusted estimate**^**3**^	**95% CI**	**p for trend**
	Estimated date of delivery based on US									
≤ 175	24/479	1.0	1.0	Ref		27/477	1.0	1.0	Ref	
176-178	26/371	1.4	1.5	0.8,2.8		29/368	1.4	1.3	0.7,2.3	
179-181	24/388	1.2	1.4	0.7,2.6		22/390	1.0	0.9	0.5,1.7	
182-185	18/430	0.8	1.0	0.5,2.1		32/416	1.4	1.2	0.7,2.1	
≥186	21/378	1.1	1.4	0.7,2.6	0.16	23/376	1.1	0.9	0.5,1.7	0.70
	Estimated date of delivery based on LMP									
≤ 175	33/609	1.0	1.0	Ref		87/555	1.0	1.0	Ref	
176-178	23/473	0.9	0.9	0.5,1.8		66/430	1.0	1.0	0.7,1.4	
179-181	27/487	1.0	1.1	0.7,2.0		54/460	0.8	0.7	0.5,1.1	
182-185	27/525	0.9	1.1	0.7,2.1		67/485	0.9	0.8	0.6,1.2	
≥186	28/484	1.1	1.2	0.8,2.2	0.24	55/357	0.8	0.7	0.5,1.1	0.11

^1^Pregnancy length according to date of delivery estimated by ultrasound (US) (2159 pregnancies) and by first day of last menstrual period (LMP) (2716 pregnancies). ^2^Adjusted for paternal age at HUNT 2 participation. ^3^Additionally adjusted for maternal height, maternal age at birth, duration between baseline ( HUNT 2 participation) and delivery, maternal education, parity, smoking, receiving social security benefits or not, pre-pregnancy maternal hypertension, diabetes mellitus, kidney and heart disease, BMI, systolic and diastolic blood pressure, blood concentration of glucose and lipids.

**Table 9:** **Crude and adjusted associations of maternal and paternal height with offspring birth weight**^**1**^

	**N**	**Age-adjusted**^**2**^	**P for trend**	**Add. adjusted**^**3**^	**95% CI**	**P for trend**	**Add. adjusted**^**4**^	**95% CI**	**P for trend**
Differences in z-score for birth weight									
Maternal height(cm), by quintiles)									
≤163	701	0		0	Ref		0	Ref	
163-165	511	0.2		0.1	0.0,0.3		0.1	0.0,0.3	
166-168	592	0.2		0.2	0.0,0.3		0.2	0.1,0.3	
169-172	589	0.3		0.2	0.1,0.3		0.2	0.1,0.4	
≥173	424	0.5	<0.0001	0.4	0.3,0.6	<0.0001	0.4	0.4,0.7	<0.0001
Paternal height (cm), by quintiles									
≤ 175	668	0		0	Ref		0	Ref	
176-178	518	0.2		0.1	0.0,0.3		0.1	0.0,0.3	
179-181	525	0.2		0.1	0.0,0.3		0.2	0.0,0.3	
182-185	582	0.3		0.2	0.1,0.3		0.2	0.1,0.4	
≥186	524	0.4	<0.0001	0.3	0.1,0.4	<0.0001	0.3	0.2,0.4	<0.0001

^1^Birth weight assessed as differences in sex- and gestational age-adjusted z-scores for birth weight and estimated in 2798 offspring of parents participating in HUNT 2 1995-1997. ^2^Adjusted for parental age at HUNT. ^3^Additionally adjusted for maternal age at birth, duration between Hunt2 and delivery, education, parity, smoking, receiving social security benefits and partner`s height ^4^Additionally adjusted for maternal cardiovascular risk factors: prepregnancy and gestational hypertensive disease and, diabetes of the mother^5^ z-score for birth weight indicate the standard deviations of offspring’s birth weight above or below the expected mean birth weight for gestational age and sex. A mean difference of z score = 0.1corresponds to a difference of 46 grams if the infant is male and delivered at a gestational age of 40.0 weeks. Thus, at a gestational age of 40.0 weeks, the offspring birth weight of a male newborn differ by 184 grams between the tallest and shortest maternal height groups and by 138 grams between the tallest and shortest paternal height groups.

Birth weight assessed as differences in sex-and gestational age –adjusted z-scores for birth weight and estimated in 2798 offspring of parents participating in the HUNT Study 1995-1997.

In separate analyses, we assessed potential effect modification between maternal height and cardiovascular risk factors, but found no consistent evidence of any interaction (all P-values above 0.10, except for HDL cholesterol with p=0.03) (results not shown).

## Discussion

We found a positive association of maternal height with pregnancy length per se, and the effect was stronger when EDD was estimated by ultrasound than by LMP. Women with shorter stature had lower risk of post-term deliveries, and when EDD was based on ultrasound, they also had higher risk of preterm births. Paternal height and common cardiovascular risk factors of the father showed no association with length of pregnancy or with the risk of pre-and post-term births.

A Norwegian study among women with low risk pregnancies, spontaneous start of delivery, and EDD estimated by LMP found no association of maternal height with length of pregnancy [4]. However, the authors of a Swedish study among 952 630 unselected pregnant women whose gestational length was estimated by ultrasound in early second trimester, reported that unadjusted mean gestational length was 2 days shorter in mothers of short stature (< 160 cm) compared to those who were taller than 160 cm [5].

We assessed paternal height and levels of paternal cardiovascular risk factors such as blood pressure, BMI, serum glucose and lipids, in relation to pregnancy length and risk of pre- and post-term birth. In contrast to previously reported associations between unfavorable cardiovascular risk factors among mothers and pregnancy length [14], no such associations were observed for the fathers. Intergenerational studies have suggested that fathers may be of importance in determining pregnancy length in term and post-term pregnancies [2,18,19], but there has been little evidence for a paternal contribution to the risk of preterm birth [24,25]. In this study, paternal height was neither associated with pregnancy length nor with the risk of pre- and post-term birth. To our knowledge, these relations have not been reported previously. In line with a recent review [26], however, we found a positive association of paternal height with offspring birth weight.

The population based prospective design of the present study makes it unlikely that selection or recall bias can explain our findings. The attendance to HUNT 2 was 71%, and in a follow-up study of 685 (2.5%) non-responders it was concluded that practical reasons such as time constraints and moving out of the county were the main reasons for young people not to attend [20]. Thus, the participants at fertile age in our study are likely to be representative for the source population. The relatively large sample size and the standardized measurements of height and other clinical measures in HUNT 2 ensure high precision of the effect estimates, and comprehensive information from self-administered questionnaires provides access to a range of possible confounders. By combining data from the HUNT 2 Study and the MBR it was possible to control for metabolic factors and other known risk factors on an individual basis. A potential limitation in this study is that smoking status was sampled before rather than during pregnancy. This was due to lack of registration of smoking status in MBR until 1999. We performed sensitivity analyses restricted to pregnancies with available information on smoking during pregnancy from 1999 to 2005, and the estimates did not differ substantially from the main results.

The MBR in Norway is a nationwide registry that includes information about virtually all births that have occurred in the country since 1967. Almost all pregnant women in Norway receive antenatal care, and hospital deliveries are free of charge, which minimizes any potential selection bias [21]. EDD was estimated by two different methods for most of the women (ultrasound and last menstrual period), and the use of both methods was standardized throughout the study period. The internal validity of our results is regarded as good. Generalization of the results to other populations must still be done with caution, since the population under study was rather homogenous with less than 3% of Caucasian women.

The LMP method is limited by inaccurate maternal recall, uncertain time of conception and implantation, irregular menses/oligomenorrea and pre-pregnancy use of hormonal contraceptives. If shorter women have higher risk of hormonal disturbances with delayed ovulation, this could have biased our findings by underestimating EDD in LMP-based analyses. Adjustment for menstrual disturbances in the statistical analyses did not, however, substantially alter the results.

It is generally agreed that ultrasound biometry in early second trimester gives a more accurate prediction of date of delivery (EDD) and reduces the rate of post-term deliveries compared to LMP dating [27]. However, the ultrasound method is based on the assumption that the size of all fetuses is similar at a given gestational age during the first half of pregnancy, whereas several studies suggest that fetal size (BPD) may differ substantially during the first half of pregnancy according to fetal sex, fetal growth restriction, and maternal smoking [28-30]. If fetal size (BPD) in early second trimester also differ according to maternal height, ultrasound dating may induce a biased estimate of EDD [31]. Femur length of the fetus at 18–19 gestational weeks has been reported to correlate with maternal height, [32]maternal height is a known determinant of offspring birth weight [5,33,34],and fetal size in early second trimester is positively associated with offspring birth weight [35,36]. Thus, it is not unlikely that ultrasound in 17–19 weeks of gestation may underestimate the true gestational age of a short woman and shift the EDD to a later date due to her smaller than average sized fetus, and vice versa for taller women. As a result, shorter women may have more severe post-term pregnancies than taller women and may therefore be at higher risk of adverse perinatal outcomes as well [31]. Taller women, on the other hand, may risk labor inductions on false post-term indications. A large population-based study reported that the replacement of LMP-dating with second trimester ultrasound dating in Sweden resulted in an increased risk of post- term perinatal morbidity and mortality for female fetuses [37]. The smaller size of female fetuses compared to males at time of ultrasound measurement most likely resulted in an underestimation of the true gestational age and more severely post term pregnancies among mothers of female fetuses. Whether the rate of post-term adverse perinatal outcomes may differ between short and tall women is not known, and unfortunately we did not have sufficient analytical power to investigate this in our data.

The observation that small fetuses grow slower and have longer pregnancies than average-sized fetuses, and vice versa for large fetuses, applies to low risk/non-pathological pregnancies for humans, and are also observed for some other mammals [2,4,38,39]. The opposite is documented for pathological pregnancies; i.e. women with slow intrauterine fetal growth have increased risk of both spontaneous and iatrogenic preterm births compared to other women [40,41]. Since maternal height is a predictor of offspring birth size [33,34] and fetal growth may influence pregnancy length, fetal growth could be an intermediate factor for the association of maternal height with gestational length. We did not have access to data on serial ultrasound measurements of fetal size to assess fetal growth. Offspring birth weight could serve as an indicator of fetal growth. However, birth weight was not regarded as a confounder, but as an intermediate factor or a common consequence of maternal height and gestational length. Thus, to avoid introducing a bias to the results, we chose not to adjust the analyses for birth weight. As an alternative approach, we separately assessed associations of parental heights with birth weight z-scores, and found that paternal height was positively associated with offspring birth weight. The association of paternal height with birth weight was of similar strength to that of maternal height, and contrasted the finding of no association between paternal height and pregnancy length. This different effect of parental height may suggest that gestational length and offspring birth weight are determined by different parental factors, and that the positive association of maternal height with gestational length cannot solely be explained by fetal growth.

Short women are at increased risk of cardiovascular disease compared to tall women, and length of pregnancy tends to be shorter in women who are at increased cardiovascular risk [14,17]. According to the fetal origins hypothesis, poor nutritional conditions *in utero* may program both slow intrauterine growth and increased risk of later cardiovascular disease [42]. If the short stature of the woman has an intrauterine origin, their higher cardiovascular risk may also have originated *in utero*, and could possibly explain the observed association of short maternal height with short gestational length. However, adjustment for maternal cardiovascular risk factors did not change the effect estimates in the present study, and is therefore an unlikely explanation of the results. Possible effect modification of cardiovascular risk factors was also assessed, but we found no evidence of any interaction. Similarly, unfavorable socioeconomic conditions of the mother could be a common cause for short maternal stature and short pregnancy length, but adjustment for socioeconomic measures did not influence the results.

In line with other research, our descriptive data suggest that shorter women experience pregnancy complications more frequently than taller women, including SGA offspring, and acute and elective Caesarian section [5,7]. Preeclampsia, stillbirth and perinatal deaths have also been reported to be associated with short stature of the mother [5,8,10]. After excluding induced births (by medication or by caesarian sections), and preeclampsia, SGA and stillbirths, the associations between maternal height and length of pregnancy became weaker in our study. This indicates that some of the association between maternal height and gestational length may be explained by a higher incidence of pregnancy complications and caesarian sections before labour among shorter women than among taller. The higher frequency of elective caesarian sections among shorter women may further reflect their higher risk of previous complications, such as previous caesarian section and/or traumatic labour experience [12,43].

We cannot rule out that the observed association of maternal height with gestational length may have some biological basis. Blacks and Asians have shorter average gestational length and higher risk of preterm birth than white Americans and Europeans, and teenage mothers have shorter length of pregnancy and higher risk of preterm birth compared to adult mothers [44,45]. A smaller or more constricted female pelvis of teenage and Asian mothers has been suggested to facilitate shorter duration of pregnancy to minimize complications from cephalopelvic disproportion. In evolutionary terms similar mechanisms may explain that shorter women benefit from shorter duration of pregnancy [44]. The population in the present study is ethnically fairly homogenous and without teenage pregnancies [20].

## Conclusion

Women with shorter stature had shorter pregnancy length and lower risk of post-term pregnancies than taller women. The associations were stronger when pregnancy length was based on ultrasound, and shorter women also had increased risk of preterm births in ultrasound-based analyses. The associations between maternal height and pregnancy length could partly be explained by a higher risk of elective caesarian sections and more pregnancy complications among shorter women. Cardiovascular risk factors did not explain the associations.

Early second trimester ultrasound is the method of choice for estimating EDD in many areas of the world and gestational age is a crucial determinant of perinatal outcome. Thus, it remains to be clarified whether the observed association between maternal height and gestational length may have any clinical consequences.

## Competing interests

There are no conflicts of interest to be declared by the authors.

All researchers are independent from funders.

## Authors’ contribution

KM and PRR had full access to all of the data in the study and take full responsibility for the integrity of the data and the accuracy of the data analysis. PRR conceived and planned the study. KM wrote the first draft of the paper and performed the statistical analyses together with PRR and EBM. LJV, KÅS, and EBM participated in the interpretation of the analyses, and the revising and writing of the article. All authors read and approved the final manuscript.

## Pre-publication history

The pre-publication history for this paper can be accessed here:

http://www.biomedcentral.com/1471-2393/13/33/prepub

## References

[B1] Clausson B Lichtenstein P Cnattingius S Genetic influence on birthweight and gestational length determined by studies in offspring of twins BJOG 2000 107 3 375 381 10.1111/j.1471-0528.2000.tb13234.x 10740335

[B2] Lie RT Wilcox AJ Skjaerven R Maternal and paternal influences on length of pregnancy Obstet Gynecol 2006 107 4 880 885 10.1097/01.AOG.0000206797.52832.36 16582127

[B3] Bergsjo P Denman DW 3rd Hoffman HJ Meirik O Duration of human singleton pregnancy. A population-based study Acta Obstet Gynecol Scand 1990 69 3 197 207 10.3109/00016349009028681 2220340

[B4] Johnsen SL Wilsgaard T Rasmussen S Hanson MA Godfrey KM Kiserud T Fetal size in the second trimester is associated with the duration of pregnancy, small fetuses having longer pregnancies BMC Pregnancy Childbirth 2008 8 25 10.1186/1471-2393-8-25 18627638 PMC2492839

[B5] Zhang X Mumford SL Cnattingius S Schisterman EF Kramer MS Reduced birthweight in short or primiparous mothers: physiological or pathological? BJOG 2010 117 10 1248 1254 10.1111/j.1471-0528.2010.02642.x 20618317 PMC3071625

[B6] Johnsen SL Rasmussen S Sollien R Kiserud T Fetal age assessment based on ultrasound head biometry and the effect of maternal and fetal factors Acta Obstet Gynecol Scand 2004 83 8 716 723 15255843 10.1111/j.0001-6349.2004.00485.x

[B7] Sherrard A Platt RW Vallerand D Usher RH Zhang X Kramer MS Maternal anthropometric risk factors for caesarean delivery before or after onset of labour BJOG 2007 114 9 1088 1096 10.1111/j.1471-0528.2007.01275.x 17617199

[B8] Sohlberg S Stephansson O Cnattingius S Wikstrom AK Maternal body mass index, height, and risks of preeclampsia Am J Hypertens 2011 25 1 120 125 21976280 10.1038/ajh.2011.175

[B9] Clausson B Cnattingius S Axelsson O Preterm and term births of small for gestational age infants: a population-based study of risk factors among nulliparous women Br J Obstet Gynaecol 1998 105 9 1011 1017 10.1111/j.1471-0528.1998.tb10266.x 9763054

[B10] Cnattingius S Haglund B Kramer MS Differences in late fetal death rates in association with determinants of small for gestational age fetuses: population based cohort study BMJ 1998 316 7143 1483 1487 10.1136/bmj.316.7143.1483 9582131 PMC28545

[B11] Chan BC Lao TT Maternal height and length of gestation: does this impact on preterm labour in Asian women? Aust N Z J Obstet Gynaecol 2009 49 4 388 392 10.1111/j.1479-828X.2009.01006.x 19694693

[B12] Sheiner E Levy A Katz M Mazor M Short stature–an independent risk factor for Cesarean delivery Eur J Obstet Gynecol Reprod Biol 2005 120 2 175 178 10.1016/j.ejogrb.2004.09.013 15925047

[B13] Kramer MS Coates AL Michoud MC Dagenais S Hamilton EF Papageorgiou A Maternal anthropometry and idiopathic preterm labor Obstet Gynecol 1995 86 5 744 748 10.1016/0029-7844(95)00267-U 7566841

[B14] Magnussen EB Vatten LJ Myklestad K Salvesen KA Romundstad PR Cardiovascular risk factors prior to conception and the length of pregnancy: population-based cohort study Am J Obstet Gynecol 2011 204 6 526 e521-528 21457914 10.1016/j.ajog.2011.02.016

[B15] Catov JM Ness RB Wellons MF Jacobs DR Roberts JM Gunderson EP Prepregnancy lipids related to preterm birth risk: the coronary artery risk development in young adults study J Clin Endocrinol Metab 2010 95 8 3711 3718 10.1210/jc.2009-2028 20501685 PMC2913035

[B16] Magnussen EB Vatten LJ Lund-Nilsen TI Salvesen KA Davey Smith G Romundstad PR Prepregnancy cardiovascular risk factors as predictors of pre-eclampsia: population based cohort study BMJ 2007 335 7627 978 10.1136/bmj.39366.416817.BE 17975256 PMC2072028

[B17] Paajanen TA Oksala NK Kuukasjarvi P Karhunen PJ Short stature is associated with coronary heart disease: a systematic review of the literature and a meta-analysis Eur Heart J 2010 31 14 1802 1809 10.1093/eurheartj/ehq155 20530501

[B18] Lunde A Melve KK Gjessing HK Skjaerven R Irgens LM Genetic and environmental influences on birth weight, birth length, head circumference, and gestational age by use of population-based parent-offspring data Am J Epidemiol 2007 165 7 734 741 10.1093/aje/kwk107 17311798

[B19] Morken NH Melve KK Skjaerven R Recurrence of prolonged and post-term gestational age across generations: maternal and paternal contribution BJOG 2011 118 13 1630 1635 10.1111/j.1471-0528.2011.03154.x 21985579

[B20] Holmen J Midthjell K Kruger Ø Langhammer A Holmen TL Bratberg G Vatten L Lund-Larsen P The Nord-Trøndelag Health Study 1995–97 (HUNT 2): Objectives, contents, methods and participation Norsk Epidemiologi 2003 13 19 32

[B21] Backe B Overutilization of antenatal care in Norway Scand J Public Health 2001 29 2 129 132 11484865

[B22] Skjaerven R Gjessing HK Bakketeig LS Birthweight by gestational age in Norway Acta Obstet Gynecol Scand 2000 79 6 440 449 10.1080/j.1600-0412.2000.079006440.x 10857867

[B23] Williams RL A note on robust variance estimation for cluster-correlated data Biometrics 2000 56 2 645 646 10.1111/j.0006-341X.2000.00645.x 10877330

[B24] Wilcox AJ Skjaerven R Lie RT Familial patterns of preterm delivery: maternal and fetal contributions Am J Epidemiol 2008 167 4 474 479 10.1093/aje/kwm319 18048376

[B25] Svensson AC Sandin S Cnattingius S Reilly M Pawitan Y Hultman CM Lichtenstein P Maternal effects for preterm birth: a genetic epidemiologic study of 630,000 families Am J Epidemiol 2009 170 11 1365 1372 10.1093/aje/kwp328 19854802

[B26] Shah PS Paternal factors and low birthweight, preterm, and small for gestational age births: a systematic review Am J Obstet Gynecol 2010 202 2 103 123 10.1016/j.ajog.2009.08.026 20113689

[B27] Gardosi J Geirsson RT Routine ultrasound is the method of choice for dating pregnancy Br J Obstet Gynaecol 1998 105 9 933 936 10.1111/j.1471-0528.1998.tb10253.x 9763041

[B28] Wald N Cuckle H Nanchahal K Turnbull AC Sex differences in fetal size early in pregnancy Br Med J (Clin Res Ed) 1986 292 6513 137 10.1136/bmj.292.6513.137 PMC1339144 3080090

[B29] Thorsell M Kaijser M Almstrom H Andolf E Expected day of delivery from ultrasound dating versus last menstrual period–obstetric outcome when dates mismatch BJOG 2008 115 5 585 589 10.1111/j.1471-0528.2008.01678.x 18333938

[B30] Persson PH Grennert L Gennser G Kullander S A study of smoking and pregnancy with special references to fetal growth Acta Obstet Gynecol Scand Suppl 1978 78 33 39 281097 10.3109/00016347809162700

[B31] Henriksen TB Wilcox AJ Hedegaard M Secher NJ Bias in studies of preterm and postterm delivery due to ultrasound assessment of gestational age Epidemiology 1995 6 5 533 537 10.1097/00001648-199509000-00012 8562631

[B32] Pierce BT Hancock EG Kovac CM Napolitano PG Hume RF Jr Calhoun BC Influence of gestational age and maternal height on fetal femur length calculations Obstet Gynecol 2001 97 5 Pt 1 742 746 11339927 10.1016/s0029-7844(01)01319-9

[B33] Mongelli M Gardosi J Longitudinal study of fetal growth in subgroups of a low-risk population Ultrasound Obstet Gynecol 1995 6 5 340 344 10.1046/j.1469-0705.1995.06050340.x 8590205

[B34] Voigt M Rochow N Jahrig K Straube S Hufnagel S Jorch G Dependence of neonatal small and large for gestational age rates on maternal height and weight–an analysis of the German perinatal survey J Perinat Med 2010 38 4 425 430 20443667 10.1515/jpm.2010.059

[B35] Smith GC Smith MF McNay MB Fleming JE First-trimester growth and the risk of low birth weight N Engl J Med 1998 339 25 1817 1822 10.1056/NEJM199812173392504 9854117

[B36] Bukowski R Smith GC Malone FD Ball RH Nyberg DA Comstock CH Hankins GD Berkowitz RL Gross SJ Dugoff L Fetal growth in early pregnancy and risk of delivering low birth weight infant: prospective cohort study BMJ 2007 334 7598 836 10.1136/bmj.39129.637917.AE 17355993 PMC1853211

[B37] Skalkidou A Kieler H Stephansson O Roos N Cnattingius S Haglund B Ultrasound pregnancy dating leads to biased perinatal morbidity and neonatal mortality among post-term-born girls Epidemiology 2010 21 6 791 796 10.1097/EDE.0b013e3181f3a660 20805749

[B38] Bourdon RM Brinks JS Genetic, environmental and phenotypic relationships among gestation length, birth weight, growth traits and age at first calving in beef cattle J Anim Sci 1982 55 3 543 553 7130063 10.2527/jas1982.553543x

[B39] Cleal JK Poore KR Newman JP Noakes DE Hanson MA Green LR The effect of maternal undernutrition in early gestation on gestation length and fetal and postnatal growth in sheep Pediatr Res 2007 62 4 422 427 10.1203/PDR.0b013e31813cbe60 17667859

[B40] Morken NH Kallen K Jacobsson B Fetal growth and onset of delivery: a nationwide population-based study of preterm infants Am J Obstet Gynecol 2006 195 1 154 161 10.1016/j.ajog.2006.01.019 16813752

[B41] Lackman F Capewell V Richardson B DaSilva O Gagnon R The risks of spontaneous preterm delivery and perinatal mortality in relation to size at birth according to fetal versus neonatal growth standards Am J Obstet Gynecol 2001 184 5 946 953 10.1067/mob.2001.111719 11303203

[B42] Barker DJ The fetal and infant origins of adult disease BMJ 1990 301 6761 1111 10.1136/bmj.301.6761.1111 2252919 PMC1664286

[B43] Kolas T Hofoss D Daltveit AK Nilsen ST Henriksen T Hager R Ingemarsson I Oian P Indications for cesarean deliveries in Norway Am J Obstet Gynecol 2003 188 4 864 870 10.1067/mob.2003.217 12712077

[B44] Patel RR Steer P Doyle P Little MP Elliott P Does gestation vary by ethnic group? a London-based study of over 122,000 pregnancies with spontaneous onset of labour Int J Epidemiol 2004 33 1 107 113 10.1093/ije/dyg238 15075154

[B45] Lao TT Ho LF The obstetric implications of teenage pregnancy Hum Reprod 1997 12 10 2303 2305 10.1093/humrep/12.10.2303 9402300

